# Palliative care stakeholders in Canada

**DOI:** 10.1186/s12961-022-00855-w

**Published:** 2022-06-06

**Authors:** Jingjie Xiao, Carleen Brenneis, Konrad Fassbender

**Affiliations:** 1 Covenant Health Palliative Institute, c/o Grey Nuns Community Hospital, Room 416 St. Marguerite Health Services Centre, 1090 Youville Drive West, Edmonton, AB T6L 0A3 Canada; 2 Department of Oncology, Faculty of Medicine and Dentistry, University of Alberta, Edmonton, AB Canada

**Keywords:** Palliative care, Stakeholders, Stakeholder analysis, Stakeholder identification, Canada

## Abstract

**Background:**

Improving access to palliative care for Canadians requires a focused collective effort towards palliative and end-of-life care advocacy and policy. However, evolution of modern palliative care in Canada has resulted in stakeholders working in isolation. Identification of stakeholders is an important step to ensure that efforts to improve palliative care are coordinated. The purpose of this analysis is to collectively identify, classify and prioritize stakeholders who made contributions to national palliative care policies in Canada.

**Methods:**

A systematic grey literature search was conducted examining policy documents (i.e. policy reports, legislative bills, judicial court cases) in the field of palliative care, end-of-life care and medical assistance in dying, at the national level, over the last two decades. Organizations’ names were extracted directly or derived from individuals’ affiliations. We then classified stakeholders using an adapted classification approach and developed an algorithm to prioritize their contributions towards the publication of these documents.

**Results:**

Over 800 organizations contributed to 115 documents (41 policy reports, 11 legislative, 63 judicial). Discussions regarding national palliative care policy over the last two decades peaked in 2016. Stakeholder organizations contributing to national palliative care policy conversations throughout this period were classified into six types broadly representative of society. The ranking algorithm identified the top 200 prioritized stakeholder organizations.

**Conclusions:**

Stakeholders from various societal sectors have contributed to national palliative care conversions over the past two decades; however, not all the stakeholder organizations engaged to the same extent. The information is useful when a need arises for increased collaboration between stakeholders and can be a starting point for developing more effective engagement strategies.

## Background

In Canada, the demographic shift to an ageing population, combined with the rising incidence of noncommunicable diseases like cancer and dementia, is increasing the demand for palliative care services [1]. Before the COVID-19 pandemic, it was estimated that the number of Canadians dying each year would increase by 40% to 330,000 by 2026 [2]. Likewise, cultural shifts are emerging, from a mindset that prioritizes curative treatments to one which values a palliative approach to care that regards dying as a normal process, and which seeks to enhance quality of life for dying patients and their families [3, 4]. Despite a national commitment to enhance palliative care across the country, the provision of palliative care in Canada remains a work in progress since its inception in the 1970s, and its availability and access are still urgent policy and practice imperatives. The government and organizations from various sectors have attempted to better understand and address the anticipated growth in demand for high-quality palliative care [4–6]. Diverse forms and types of information and communication have arisen to inform public policy on palliative care.

Effective policies have proved to yield tangible results at a national level in other jurisdictions. For example, the launch of Spain’s national strategy led to a 50% increase in palliative care teams and unified regional approach [7]. Likewise, in Canada, policy interventions to improve the quality of death through the provision of high-quality palliative care have gained momentum in recent years. Health Canada first established the Secretariat on Palliative and End-of-Life Care (June 2001) and hosted the National Action Planning Workshop on End-of-Life Care (March 2002). Five working groups were then established to address the priority areas identified for action [8]. A foundational report for continued work to enhance Canada's capacity for quality and accessible palliative and end-of-life care was published in 2007 [9]. Ten years later, the federal government passed a bill (Bill C-277) to create a framework for palliative care in Canada. Health Canada subsequently launched a broad, multipronged consultation process, designed to reach Canadians, healthcare providers, caregivers, people living with life-limiting illness and subject matter experts.

The Framework on Palliative Care in Canada was published in December 2018, which “reflects the voices of the many Canadians heard throughout the consultations and serves as a guideline for all palliative care stakeholders to use to improve access across Canada” [10]. Additionally, a consequent evaluation of the framework is scheduled to be completed within 5 years after its release. The framework functions as an overarching guideline; however, palliative care services and programmes are not provided consistently across both geography and time [10]. In Canada, there are 14 different systems in place for providing care (13 provincial/territorial jurisdictions in addition to the federal government, which has responsibility for mandated populations). Considerable variation and disparity in palliative care service delivery exist across Canada as a result of the differing regional demographics, societal needs and funding structures. Coordinating efforts towards the implementation and evaluation of the framework requires an understanding of stakeholders and their incentives. In this analysis we identify who is affected by the framework and who has the power to influence its implementation (i.e. stakeholders).

The term stakeholders mentioned herein refers to organizations, groups of persons or individuals who influence or are influenced by choices and regulations by another organization [11]. A stakeholder analysis process consists of systematically gathering and analysing qualitative information to determine whose interests should be taken into account when developing and/or implementing a policy or programme [12]. In this study, we employ a systematic approach to identify and prioritize stakeholders based on their contributions to national palliative care policies in Canada.

## Methods

### Study framework

This stakeholder identification study employed an environmental scan of the grey literature. We adopted and modified the first three (out of eight) steps of a stakeholder analysis method developed by Kammi Schmeer, which is part of the Policy Toolkit for Strengthening Health Sector Reform [12]. The Schmeer guideline provides instructions and tools that are supported by both academic theory and real-world application [12]. This kind of stakeholder analysis is designed to help policy-makers, managers and their working groups systematically collect and analyse data about key health reform stakeholders. Building on the first three steps, we developed a grey literature search and stakeholder identification method in order to understand who the palliative care stakeholders are and what roles they play in conducting palliative care policies at a national level in Canada.

### Grey literature search

We conducted a systematic grey literature search for consultative reports and legislative and judicial proceedings in the field of palliative care, end-of-life, and medical assistance in dying (MAID). By definition, grey literature refers to literature “produced on all levels of government, academics, business and industry in print and electronic formats, but which is not controlled by commercial publishers” [13]. We used web search for grey literature because reports, white papers or working papers created by governments, advocacy organizations or other organizations are typically disseminated on the Internet rather than as published, peer-reviewed scholarly journal articles [14, 15].

In the current study, consultative reports at a national level were identified by searching the PsycExtra [16], AMICUS [17], Voilà [18] and Google databases. These databases provide access to unpublished or grey literature which covers content outside the peer review system, such as guidelines, standards, technical reports and proceedings. The following keywords were searched using the “Any field” search box: palliative, end-of-life, care AND palliative OR end-of-life, supportive care, comfort care, advance care planning, medical assistance in dying, assisted death, MAID. Search results were date-limited from 1 January 1995 to 31 December 2018. Documents compiled by the Palliative Care Matters initiative were used as a supplementary resource [19].

Our grey literature review included legal documents, including legislative bills and judicial court cases relevant to palliative care, end-of-life care or MAID (Table 1). The LEGISinfo [20] database was searched for legislative bills. For judicial documents, we searched the End-of-Life Law and Policy in Canada database maintained by Dr Jocelyn Downie from the Health Law Institute at Dalhousie University (dated to 31 December 2018). This database provides a comprehensive and up-to-date list of court cases with respect to palliative, end-of-life and MAID issues [21].

**Table 1:** Inclusion and exclusion criteria for national palliative care documents

Document type	Inclusion criteria	Exclusion criteria
Reports	•Sponsored or authored by Canadian governments (national, provincial and regional), health authority or other organizations in Canada •Significant focus on palliative care, including policy and/or recommendations •Published between January 1995 and December 2018	•Documents focused on a single disease with little palliative care content •Regional reports •Annual reports •Research reports •Literature reviews •Clinical practice guidelines •Progress reports
Legislative documents	•Canadian federal, provincial and territorial statutes, bills, regulations, debates and orders-in-council •Significant focus on palliative care, including policy and/or recommendations •Published between January 1995 and December 2018	•Documents with little palliative care content
Judicial court cases	•Court cases focusing on palliative interventions (e.g. potentially life-shortening symptom relief and palliative sedation), the withholding and withdrawal of potentially life-sustaining treatment, advance directives, assisted suicide and euthanasia •Significant focus on palliative care, including policy and/or recommendations •Published between January 1990 and December 2018	•Documents with little palliative care content

The inclusion and exclusion criteria described in Table 1 were used to further reduce the number of search results for national palliative care documents**.**

### Data extraction

From policy documents identified in grey literature, organizations who made contributions to national publications were extracted: directly or indirectly. Organizations who engaged significantly as one entity were abstracted directly. Organizations were also derived indirectly from the affiliations of individuals who engaged significantly in these documents. We excluded stakeholder identification at the individual level when they were not affiliated to any organization.

Regarding the contributions made by organizations or individuals, we defined eight roles, which classified organizations’ contributions according to their efforts towards publication of the national policy documents. For authorship, we adopted the International Committee of Medical Journal Editors definition and its four criteria [22]. We defined sponsor as the organization who commissioned the report and funder as the organization who provided funding for the report. Notably, we assumed that the major cost was the research and writing of the report. Given that the process of gathering evidence required operational oversight and additional effort, organizations or individuals who acted as chair, cochair, project lead, operational managers or committee cofounders were classified as lead. Nonacademic researchers and consultants referred to those whose role was to generate evidence, for example, conducting systematic review. Organizations and/or individuals who contributed to providing evidence were categorized as contributors, including advisory/steering/planning/coordinating/standards committee members, project task group members, participants/attendants at interviews or roundtables, expert consultants, town hall/panellist/panel speakers, and featured organizations/physician leaders/palliative care delivery models. Witness and intervenor were used to define specialized organizational roles in legislative bills and judicial court cases, respectively. The role of organizations and individuals was an indicator of their engagement. Significant roles referred to organizations and/or individuals who contributed towards the national documents as authors, sponsors and/or funders.

The extraction of the above information (i.e. stakeholders and their roles) assumed that the contribution of an organization or individual was relevant to palliative care in nature and involved consultative processes. Specific criteria for the organizations or the organizational affiliations of individuals are shown in Table 2.

**Table 2:** Inclusion and exclusion criteria for organizations contributing to eligible national palliative care documents

Organizations identified from the following documents	Inclusion criteria	Exclusion criteria
Reports	Named organizations For-profit and not-for-profit corporations, including charities and foundations Governmental organizations Member-benefit professional associations, designation-granting associations, certifying bodies and professional regulatory bodies Named collaborations, committees, working groups, collectives and other groups of individuals or organizations	Organizations that focused only on euthanasia or MAID without a mandate on advocating for palliative care Universities Organizations that had ceased to exist International organizations
Legislative documents	Named organizations that had acted as witnesses	Organizations that focused only on euthanasia or MAID without a mandate on advocating for palliative care
Judicial court cases	Named organizations that had acted as intervenors	Organizations that focused only on euthanasia or MAID without a mandate on advocating for palliative care

### Stakeholder classification

We classified organizations into groupings by adapting the stakeholder classification method from Schiller et al. [23]. This method was developed from a strategic and focused literature search with attention to categories of health stakeholders. Organizational websites were used as the primary source to evaluate their categories. Associations Canada [24], a directory which provides detailed listings and abstracts, including organizational types, for nearly 20,000 regional, national and international organizations in Canada, was used as a second source to refine and validate the first level of classification.

### Stakeholder prioritization

We created a simple, arithmetic index quantifying the nature and frequency of stakeholders’ contributions to national policy publications. This index reflects stakeholders’ engagement level and was used to prioritize a list of the top 200 stakeholders. Firstly, we calculated the rate at which each organization appeared or was repeated in the policy documents, and designated it Index 1 simple frequency. We also generated the number of policy documents in which each organization was involved as Index 2 (i.e. reports). Additionally, we sorted the frequency of significant roles (i.e. author, sponsor or funder) that each organization played in the policy documents and designated it Index 3 (i.e. significant roles). Supplemental to Index 3, we generated Index 4 to indicate the number of distinct policy documents in which an organization played a significant role (i.e. reports with significant roles). A simple summation of these four indices was used to rank the stakeholders’ engagement level. This ranking method focused primarily on quantifying stakeholders’ involvement in national palliative care policy documents on the premise that contributions could be made by any societal sector. Therefore, the organizational type was not included as an index of this ranking method.

### Ethical considerations

This is a retrospective review study. Ethics approval was obtained from the University of Alberta Research Ethics Office (Pro00090814).

## Results

**Fig. 1:**
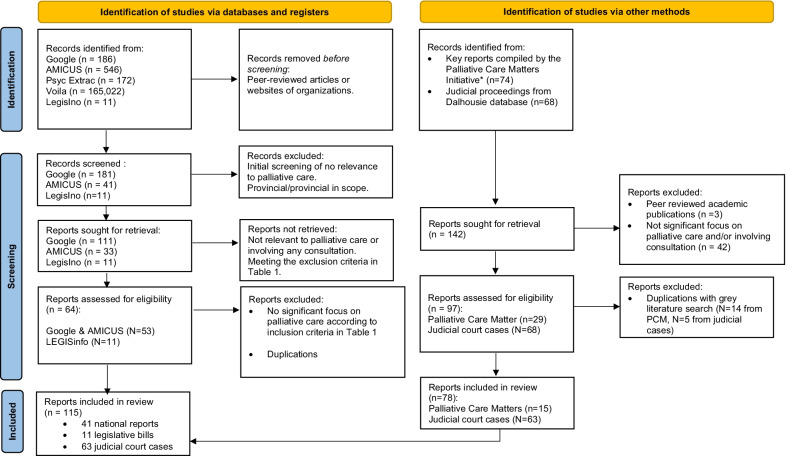
PRISMA [Preferred Reporting Items for Systematic Reviews and Meta-Analyses] flow diagram. Adapted from: http://www.prisma-statement.org/. *Reference [19]

**Fig. 2:**
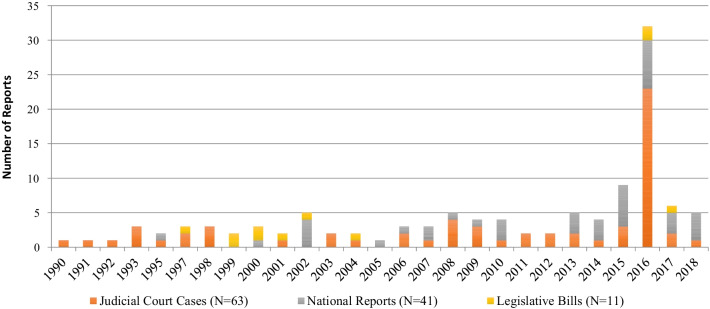
Systematic review of reports influencing palliative care policy in Canada

The grey literature search for national reports resulted in a full-text review of 111 publications, of which 53 publications were included after initial assessment for relevance and rigour (i.e. palliative care content and consultative process). Additionally, an exploratory search of reports compiled by the Palliative Care Matters initiative resulted in 15 references. A total of 68 records were further evaluated by removing duplicates and utilizing the identified inclusion/exclusion criteria, and 41 national reports were found to be eligible for inclusion. In addition, 11 legislative bills and 63 judicial court cases were identified. A flowchart of the search and identification process is shown in Fig. 1. National discussions regarding palliative care policies over the last two decades peaked in 2016, consisting of seven national reports, two legislative bills and 23 judicial court cases (Fig. 2).

### Identified stakeholders

A total of 821 distinct organizations who made 2276 contributions through 10 various roles were identified (Table 3). There were 130 significant contributions across 80 organizations.

**Table 3:** Contributions from organizations

Roles	Contributions	Organizations
Author	45	26
Sponsor	51	31
Funder	34	23
Editorial	2	1
Review	42	33
Lead	38	18
Research	8	2
Contributor	1019	482
Witness	926	422
Intervenor	108	52
Total	*n* = 2276	*n* = 821
Significant roles^a^	*n* = 130 (5.7%)	*n* = 80 (9.7%)

^a^These include author, sponsor and funder. Percentages reflect the proportion of the total number of contributions or organizations

### Stakeholder classification

**Fig. 3:**
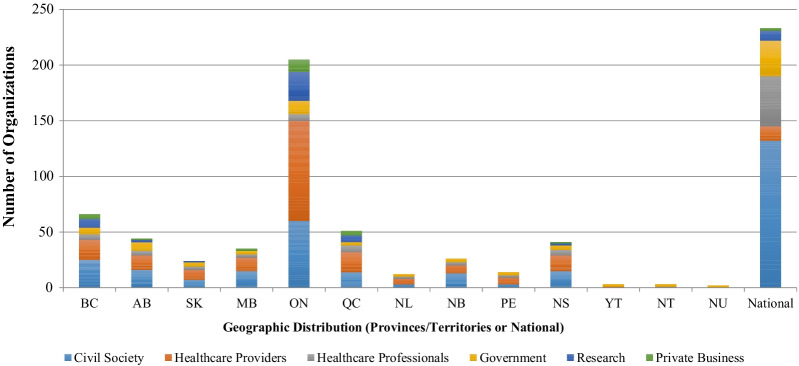
Organization distribution by types and geographical locations (*N* = 759*). BC: British Columbia; AB: Alberta; SK: Saskatchewan; MB: Manitoba; ON: Ontario; QC: Quebec; NL: Newfoundland and Labrador; NB: New Brunswick; PE: Prince Edward Island; NS: Nova Scotia; YT: Yukon; NT: Northwest Territories; NU: Nunavut. *62 out of the 821 organizations are miscellaneous and therefore not included in the figure

Six organizational groupings were identified. As shown in Table 4, most stakeholders were in the category of civil societies (*n* = 329), followed by healthcare providers (*n* = 212). An equal number of policy-makers/governments and healthcare professionals were found (*n* = 86 respectively). A relatively smaller number of stakeholders were in the category of research (*n* = 60) or private business (*n* = 31). When classifying stakeholders by their headquarter locations, 31% of them were in Ottawa at a national level, followed by 27% in Ontario. The remaining 42% were located across nine other provinces and three territories (Fig. 3).

**Table 4:** Classification of 804 stakeholder organizations^a^

Policy-makers and governments (*N* = 86)	Civil societies (*N* = 329)	Healthcare providers (*N* = 212)
Federal government Federal agencies Provincial governments Provincial agencies	First Nations Business societies Caregivers and volunteers Disability societies Disease-specific societies Funders Francophone societies Faith-based organizations Gender-based societies Justice-based societies Human rights societies Health promotion societies Palliative care societies Seniors organizations Suicide prevention societies Miscellaneous civil societies	Regional health authorities (including cancer control) Hospitals (including integrated services) Hospices Long-term care providers Community care providers Home care providers Palliative programme providers
Healthcare professionals (*N* = 86)	Private business (*N* = 31)	Research (*N* = 60)
National Provincial Miscellaneous healthcare professionals	Private businesses Consultants	Think tanks Polling firms Research projects

^a^Not including 17 organizations that did not fit into any one of these categories and thus were coded as miscellaneous

### Stakeholder prioritization

Index 1 (simple frequency) identified a wide range of contributions from a minimum of one up to the highest number of contributions made by Health Canada (*n* = 107), which was followed by the Senate of Canada (*N* = 71). Index 2 (number of reports) ranged from 1 to 24, with the Canadian Hospice Palliative Care Association (CHPCA) contributing to 24 distinct national publications, and CHPCA was followed by the Canadian Medical Association (*N* = 20). The highest number of significant roles, shown as Index 3, was 18 from Health Canada; CHPCA ranked second, with 12 significant roles. The corresponding distinct number of reports in which Health Canada and CHPCA played a significant role also ranked the highest, both in nine policy documents. The sum of the four indices ranged from 2 to 153. Consequently, 821 organizations were ranked in the order of their summed scores and a list of the top 200 stakeholder organizations was generated. The 17 organizations that did not fit into any of the stakeholder categories universally made little impact on palliative policy documents, reflected by their low summative index scores and rankings (data not shown).

## Discussion

This study identified palliative care stakeholders in Canada using a systematic framework. By examining the stakeholders involved in national policy conversations and the extent to which stakeholders contributed to policy documents, this study helps provide a better understanding of the palliative care landscape in Canada for policy-makers, administrators and organizations who have a stake in palliative care. Furthermore, the findings can help guide future work when investigating stakeholders’ characteristics and creating work plans for stakeholder engagement. The systematic approach hereby developed proved to be effective and resulted in a robust inventory of 821 stakeholders. Organizations produce their own publications mainly to provide an evidence base for policy or practice, and to inform public policy or practice [26]. We found that reports constituted most of the identified policy documents, which is consistent with findings from a previous survey with producing organizations, research users and collection services conducted in Australia [25, 26]. This survey found that 93% of organizations considered reports an important or very important publishing approach for grey literature [26]. Additionally, quality control is common during the production of this type of document [26]. Ninety percent of organizations often undertook basic copy editing and formatting in-house, and approximately 60% often had their publications reviewed by an internal board, advisory group or peer review [26]. In contrast to most previous publications, focused on either a specific setting of palliative care [27] or only certain types of documents [28], our review of the grey literature was comprehensive and comprised a variety of resources, from guiding documents to written laws and court cases. Our search represents a more comprehensive review of policy documents in the contemporary Canadian context.

Palliative care policy has been largely influenced by the introduction of MAID legislation; however, the impact was brief. The peak of national publications in 2016 was driven primarily by judicial court cases, which can likely be explained by the legal change on MAID in Canadian society and its subsequent influence on the provision of palliative and end-of-life care. MAID was decriminalized by the Canadian Supreme Court on 6 February 2016 [29]. Following the legalization, a bill was passed by parliament on 17 June 2016 which specified the conditions under which MAID could be legally provided [30]. The spirit of the law is that this new type of service is to be provided compassionately and ethically. Although this law resolves the long-running, contentious debate in Canada about the permissibility of assisted dying [31], much ambiguity remains regarding the implementation of MAID [32]. Because of the ambiguity, MAID has drawn attention to the current gaps and inconsistencies in the availability of palliative care, and created an opportunity for palliative care stakeholders to identify themselves. Therefore, the high number of court cases focusing on palliative care interventions, the withholding and withdrawal of potentially life-sustaining treatment, advance directives, assisted suicide and euthanasia during the period of this historic change is not surprising. On the other hand, the relatively small number of national policy documents in years other than 2016 indicate that efforts to capitalize on national attention to palliative care and/or MAID may have been short-lived.

Stakeholder organizations contributing to national palliative care policy conversations over the past two decades were broadly representative of society. Of note, civil society and healthcare providers rather than governments were driving palliative care conversations in the country. Despite this finding, we acknowledge that the governments and policy-makers had a unique role compared to stakeholders from other categories because of their dual responsibilities of participating in policy conversation and being accountable for the ultimate policy outcome. Of the 821 stakeholders, some of the highly ranked organizations made significant contributions, as expected, because of their continuous participation in national policy work and collaboration with the federal government over the years. For example, in the 2018 Framework on Palliative Care, 14 organizations were acknowledged as key stakeholders who had a direct role in developing foundational documents and frameworks and leading palliative care initiatives in Canada [10]. These organizations also ranked highly in the current analysis, ranging from 2 to 47 (data not shown). Although our finding confirms the contributions of these key stakeholders, this analysis identified additional stakeholders who played important roles in national palliative care conversations. These additional organizations represented a variety of societal sectors across the country.

These findings validate the need for a systematic stakeholder analysis approach, through which one can avoid the danger that particularly powerful and well-connected stakeholders can have greater visibility than more marginalized groups [33]. The distribution of identified stakeholders across all 13 provinces/territories further reinforces the requirement for collective effort, regardless of stakeholder size and region. The resulting stakeholder inventory should greatly aid in the next step to the development and implementation of stakeholder engagement strategies. In a companion paper of this stakeholder analysis, we further surveyed the identified stakeholders to understand the facilitators and barriers to working more collaboratively across Canada [34].

### Limitations

Despite the novelty of creating a systematic stakeholder identification framework, several limitations should be considered when interpreting the findings. Firstly, not all stakeholders influence policy through participation in national policy documents. Contributions through other political and public avenues, such as traditional media, web influence, letters to politicians, and working behind the scenes and operations, are not included. Secondly, stakeholders may not be appropriately acknowledged in the policy documents. This is especially true when attributing individuals’ contributions to organizations, because individuals often represent more than one organization. Thirdly, it is possible that not all national documents were indexed and available for the search. Lastly, provincial policies often serve as exemplars and influence national policy. The exclusion of provincial documents from the grey literature search may have resulted in the exclusion of important stakeholder organizations.

## Conclusions

The palliative care stakeholders identified in this study can be used by researchers, policy-makers and healthcare providers to inform productive engagement strategies and help them work more effectively, collaboratively and efficiently. In the next phase of our work, we will adapt the next four steps of the Kammi Schmeer stakeholder analysis method (i.e. adapting the tools, collecting and recording the information, filling in the stakeholder table, analysing the stakeholder table) [12] and use the findings to further understand key stakeholders’ characteristics.

## Data Availability

Full access to original data and the stakeholder inventory can be obtained by contacting the corresponding author.

## Abbreviations

MAID: Medical assistance in dying
COVID-19: Coronavirus disease
